# Prevalence of type-specific HPV among female university students from northern Brazil

**DOI:** 10.1186/s13027-015-0017-x

**Published:** 2015-07-22

**Authors:** Rodrigo Covre Vieira, Jeniffer do Socorro Valente Monteiro, Estéfane Primo Manso, Maria Renata Mendonça dos Santos, Mihoko Yamamoto Tsutsumi, Edna Aoba Yassui Ishikawa, Stephen Francis Ferrari, Karla Valéria Batista Lima, Maísa Silva de Sousa

**Affiliations:** Laboratory of Molecular and Cellular Biology, Center for Tropical Medicine (1st Floor), Federal University of Pará, Belém, Pará Brazil; College of Pharmaceutical Sciences, Federal University of Pará, Belém, Pará Brazil; Postgraduate Program in Clinical Analysis, Federal University of Pará, Belém, Pará Brazil; Cytology Laboratory, Institute of Biological Sciences, Federal University of Pará, Belém, Pará Brazil; Department of Ecology, Universidade Federal de Sergipe, Sergipe, Brazil; Bacteriology Section, Evandro Chagas Institute, Ananindeua, Pará Brazil

**Keywords:** Human papillomavirus, Prevalence, HPV vaccine impact, Genotype distribution

## Abstract

**Background:**

Human papillomavirus (HPV) infection is associated with cervical cancer, the most frequent cancer in women from northern Brazil. Assessment of the short-term impact of HPV vaccination depends on the availability of data on the prevalence of type-specific HPV in young women in the pre-immunization period, although these data are currently unavailable for the study region. The aim of this study was to estimate the distribution of all mucosal HPV genotypes, including low- and high-risk HPV types, in unvaccinated college students from northern Brazil.

**Findings:**

Specimens were collected from 265 university students during routine cervical cancer screening. The HPV DNA was assessed by Polymerase Chain Reaction and positive samples were genotyped by Restriction Fragment Length Polymorphism. Most students (85.7 %) had normal cytological results. The prevalence of HPV was 25.3 % (67/265), with a high frequency of multiple infections and non-vaccine high-risk HPV genotypes. The most prevalent type was HPV-61 (5.3 %), followed by types 82, 16, 59, and 6. Multiple infections were associated with high-risk and possibly high-risk HPVs.

**Conclusions:**

We demonstrated a high prevalence of HPV infection in university students from northern Brazil. Vaccine high-risk types were relatively rare, emphasizing the predominance of carcinogenic genotypes that are not prevented by the currently available vaccines. Our study highlights the need to reinforce cytological screening in women from northern Brazil, and promote the early diagnosis and treatment of the precancerous lesions associated with cervical cancer.

## Findings

### Introduction

Human papillomavirus (HPV) is a non-enveloped double-stranded DNA virus of the family Papillomaviridae [1]. Persistent infections with high-risk HPVs (hrHPV) can progress to Cervical Cancer (CC) [2]. While this cancer is the third most common in women both worldwide [3], and in Brazil in general, it is the most frequent in women from northern Brazil. It thus constitutes a serious public health problem in this region [4].

Prophylactic HPV vaccination in Brazil began in March 2014 as part of national immunization program for girls aged 9 to 13 years old. The quadrivalent vaccine (Gardasil) adopted for this program provides protection from the two hrHPVs found in 70 % of all cases of CC (HPV-16 and HPV-18), as well as protection from two low-risk HPVs (lrHPV) that cause about 90 % of cases of genital warts (HPV-6 and HPV-11) [5].

The effects of HPV vaccination on CC rates will only be apparent after a number of years. A short-term assessment of the impact of the vaccination program can be provided by the evaluation of the prevalence of type-specific HPV in vaccinated young women [6, 7], although the systematic evaluation of any change in the distribution of the virus will depend on the availability of data from the pre-immunization period, which are currently unavailable for the study region.

The present study aims to estimate the prevalence of all mucosal HPV types, including low-risk and hrHPV types, in female university students from northern Brazil prior to the introduction of HPV vaccination.

## Methods

Between October 2012 and October 2013, cervical specimens were collected from female students as part of a routine CC screening program based on the Pap test and analyzed at the Cytology Laboratory of the Federal University of Pará (UFPA) in Belém, Brazil. After sample collection, the endocervical brush was washed into microtubes containing 0.5 mL of saline that were stored at −20 °C until the molecular analysis. At the UFPA Molecular and Cellular Biology Laboratory, the viral DNA was extracted using the standard phenol-chloroform protocol and purified by ethanol precipitation [8]. Polymerase Chain Reaction (PCR) of the human β-globin gene was conducted prior to HPV detection to confirm the suitability of the samples [9].

The HPV PCR was conducted using degenerate MY09/MY11 primers [10] and the HPV-positive cases were typed by Restriction Fragment Length Polymorphisms (RFLP) [11], which permit the identification of the 13 hrHPVs classified by the International Agency for Research on Cancer (IARC) as being at least “probably carcinogenic to humans” (HPV 16, 18, 31, 33, 35, 39, 45, 51, 52, 56, 58, 59 and 68), twelve possibly high-risk types (HPV 26, 30, 34, 53, 66, 67, 69,70, 73, 82, 85 and 97) and other genotypes classified as lrHPVs, including HPV-6 and HPV-11 [12]. The conventional cytology results were classified according to the Bethesda's terminology [13].

This study was approved by the Human Research Ethics Committee of the UFPA Center for Tropical Medicine (n° 167.270/2012), in accordance with all the directives and norms regulating research involving humans in Resolution n°. 446/2012 of the Brazilian National Health Council. Informed consent was obtained from all participants.

## Results

A total of 265 university students were included in the study (mean age 25 ± 5.7 years; range: from 18 to 55 years). The mean age of first intercourse was 18 ± 2.9 years, and the mean age at menarche was 12 ± 1.5 years. Most (75 %) of the individuals had had two or more sexual partners during their lifetime, 90 % were single, 99 % did not smoke, and more than half (59 %) used condoms during sexual relations. Just under half (49 %) of the participants were having Pap screening for the first time.

No evidence was found of invasive carcinoma in any of the samples.

High-grade Squamous Intraepithelial Lesions (HSIL) were found in six samples (2.3 %), and Low-grade lesions (LSIL) in 17 (6.4 %), Atypical Squamous Cells of Undetermined Significance (ASCUS) in 14 (5.3 %), and Atypical Squamous Cells, cannot exclude HSIL (ASCH) in one (0.4 %). The other 227 samples analyzed in this study presented normal cytology (85.7 %).

The DNA of HPV was detected in cervical specimens of 67 women, with an overall prevalence of 25.3 % (67/265). The prevalence of HPV among women with normal cytology was 23.4 % (53/227), increasing to 36.9 % (14/38) in women with abnormal cytology of any kind. There was no statistical association between the occurrence of HPV infections and abnormal cytology (p > 0.05).

**Fig. 1:**
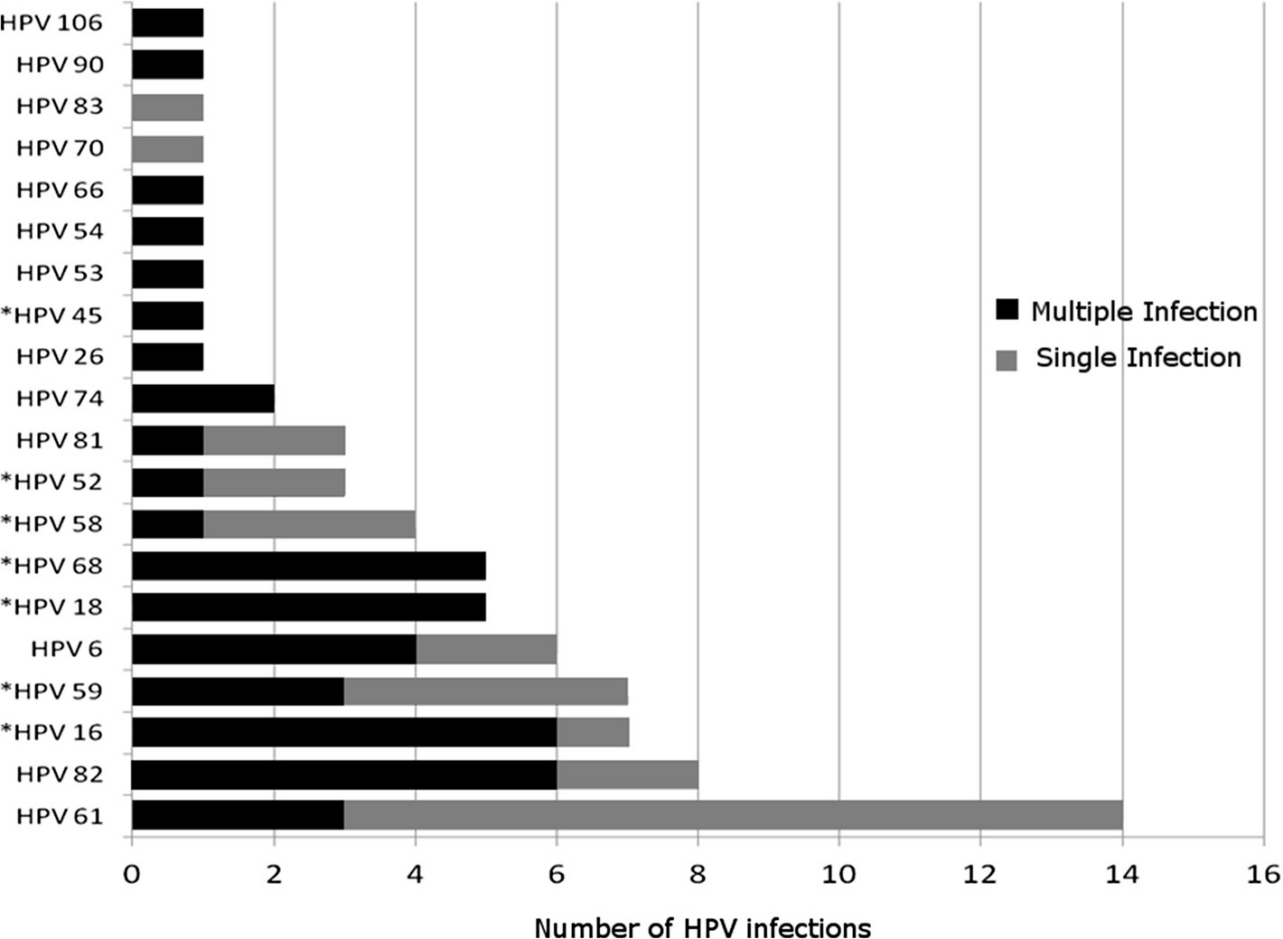
Absolute frequency of the 20 types of HPV detected in single and multiple infections (HPV: human papillomavirus; *oncogenic types)

Twenty HPV types were detected (Fig. 1), including seven hrHPVs (16, 18, 45, 52, 58, 59 and 68), five possibly hrHPVs (26, 53, 66, 70 and 82), and eight lrHPVs (6, 54, 61, 74, 81, 83, 90 and 106), with no genotype being determined in six samples. The most frequent genotype was HPV-61, followed by HPV-82, HPV-16, HPV-59 and HPV-6. Of the high-risk infections, HPV-16 and HPV-59 were the most prevalent genotypes, which were each detected in seven samples (2.7 %), whereas HPV-61 was the low-risk genotype most often detected (5.3 %). While HPV-6 was identified in six samples (2.3 %), HPV-11 was not detected at all in the present study. The possibly hrHPVs were observed in 11 samples, of which HPV-82 was the most prevalent genotype (3 %).

Single infections were detected in 59 % (36/61) of the genotyped samples, while multiple genotypes were identified in 41 % (25/61). The five most common genotypes in multiple infections were HPV-82, HPV-16, HPV-18, HPV-68 and HPV-6. High-risk HPVs and possibly hrHPVs were found more frequently in multiple infections than in single infections. Low-risk genotypes were associated with single infections (Table 1).

**Table 1:** Distribution of HPV types according to the mode of infection (single or multiple)

Type of HPV	Number of samples	Single infection (%)	Multiple Infection^a^ (%)	p*	Odds Ratio (95 % CI)^b^
hrHPV	26	9 (34.6)	17 (65.4)	0.0021	6.38 (2.06-19.72)
possibly hrHPV	11	3 (27.3)	8 (72.7)	0.0428	5.18 (1.21-22)
lrHPV	33	24 (72.7)	9 (27.3)	0.0355	0.28 (0.1-0.82)

*Values of less than 0.05 were considered significant

^a^2 or 3 different genotypes

^b^CI = Confidence Interval

High-risk HPV infections were identified in 26 samples (9.8 %), of which 12 presented vaccine HPV-16 or HPV-18 (4.5 %). Overall, non-vaccine hrHPVs were found in approximately 77 % of the high-risk cases (20/26). Fourteen individuals were infected exclusively by non-vaccine hrHPVs and six were co-infected with HPV-16 or HPV-18. Infections associated exclusively with vaccine hrHPVs were recorded in only six cases.

## Discussion

Our study demonstrated a HPV prevalence of 25.3 % among unimmunized university students from northern Brazil. In the Pap test, 85.7 % of the students screened had normal cytology. Despite the high prevalence of HPV infection, a low prevalence of vaccine hrHPVs was detected (4.5 %), with non-vaccine types predominating in high-risk cases. In addition, some HPVs that present vaccine cross-protection in vaccine trials due to their genetic similarities with HPV-16 and HPV-18, mainly HPV-31, 33, 45, 52 and 58 [14, 15], were also rare in our study, as were the vaccine lrHPVs (HPV-6 and HPV-11).

The prevalence of HPVs recorded in the present study was much higher than that found by Bruni et al. [16] in a meta-analysis of the worldwide data, in which the overall HPV prevalence among women with normal cytology was 11.7 %. In Brazil, there is considerable variation in the prevalence of HPVs and the distribution of genotypes. The prevalence recorded here was much higher than that found in previous studies in southern (6.7 %) [17] and southeastern Brazil (11 %) [18], but lower than that described in a previous study in northern Brazil (29.4 %) [19]. However, all these previous studies recorded HPV-16 as the most prevalent genotype, whereas in the present study, HPV-61 and HPV-82 were the most common, although HPV-16 was the most frequent hrHPV genotype, together with HPV-59.

The effectiveness of HPV vaccines for the reduction of the incidence of CC will depend on a number of factors, including vaccine coverage, the efficacy achieved with one, two or three doses of the vaccine, the degree of cross-protection provided against HPV types not included in the vaccines, and the prevalence of the genotypes for which the vaccines do provide protection [15]. Our study demonstrated a high prevalence of hrHPVs that are not prevented by vaccination. Despite the good vaccination coverage achieved during the first vaccination campaign, coverage for the second dose fell to 42.2 % in Brazil as a whole, and only 18.1 % in Pará, where our study was conducted [20].

Multiple infections were very common in our study, and typically involved hrHPVs and possibly hrHPV genotypes. An association may exist between some pairs of high-risk genotypes, which may determine their role in multiple infections [21]. A better understanding of the mechanisms of synergism or antagonism involved in high-risk infections will be important for the prediction of the impact of vaccination programs on the distribution and replacement of HPV genotypes [22]. The high frequency of infections by possibly hrHPVs, especially HPV-82 (the second most prevalent in our study), also emphasizes the need for stricter epidemiological surveillance of these types in northern Brazil, in addition to those already classified as hrHPVs.

The small sample size and the convenience sampling procedure adopted in our study represent two potential limitations, although these considerations are outweighed by the need for data on the prevalence and distribution of HPV genotypes from this poorly-studied region, which are essential for epidemiological monitoring and in particular, the short-term evaluation of the effectiveness of the vaccination program established recently in Brazil. The inclusion of subjects with abnormal cytology may also have been a limiting factor. Nevertheless, the future evaluation of the vaccine effectiveness requires pre-vaccination data both in women with normal and abnormal cytology results. Moreover, our study sample was not obtained from a referral centre or colposcopy clinic and only a small proportion of the participants had abnormal cytology, which did not consistently affect the overall HPV prevalence.

## Conclusions

Despite the high prevalence of HPV (25.3 %) in the study group from northern Brazil, the vaccine hrHPVs were relatively rare. In other words, there was a predominance of carcinogenic genotypes against which the vaccine does not confer immunity. This highlights the need to reinforce cytological screening as a basic tool for the early diagnosis and treatment of precancerous CC lesions in northern Brazil, where CC incidence and mortality rates are unusually high.

## Abbreviations

HPV: Human papillomavirus
hrHPV: High-risk HPV
PCR: Polymerase chain reaction
RFLP: Restriction fragment length polymorphism
lrHPV: Low-risk HPV
OR: Odds ratio
95 % CI: 95 % confidence interval
LSIL: Low-grade Squamous Intraepithelial Lesion
HSIL: High-grade Squamous Intraepithelial Lesion
ASCUS: Atypical Squamous Cells of Undetermined Significance
ASCH: Atypical Squamous Cells, cannot exclude HSIL
